# Supplementary material for: Macroscopic tunneling probe of Moiré spin textures in twisted CrI3
Source: Nat Commun. 2024 Jun 11;15:4982. doi: 10.1038/s41467-024-49261-6 (PMC11167019; doi:10.1038/s41467-024-49261-6)
Supplement: Supplementary file 1 — Supplementary Information [file 41467_2024_49261_MOESM1_ESM.pdf]

Supplementary Information for

Macroscopic Tunneling Probe of Moiré Spin Textures in Twisted CrI_3

Bowen Yang[1,2†], Tarun Patel[1,2†], Meixin Cheng[1,3], Kostyantyn Pichugin[3], Lin Tian[1,4], Nachiket Sherlekar[1,2], Shaohua Yan[5], Yang Fu[5], Shangjie Tian[5,6], Hechang Lei[5], Michael E. Reimer[1,4], Junichi Okamoto[7,8], Adam W. Tsen[1,3]*

[1]Institute for Quantum Computing, University of Waterloo, Waterloo, ON N2L 3G1, Canada.

[2]Department of Physics and Astronomy, University of Waterloo, Waterloo, ON N2L 3G1, Canada.

[3]Department of Chemistry, University of Waterloo, Waterloo, ON N2L 3G1, Canada.

[4]Department of Electrical and Computer Engineering, University of Waterloo, Waterloo, N2L 3G1, Canada.

[5]Department of Physics and Beijing Key Laboratory of Optoelectronic Functional Materials & Micro-nano Devices, Renmin University of China, 100872 Beijing, China.

[6]School of Materials Science and Engineering, Anhui University, 230601 Hefei, China

[7]Institute of Physics, University of Freiburg, Hermann-Herder-Str. 3, 79104 Freiburg, Germany.

[8]EUCOR Centre for Quantum Science and Quantum Computing, University of Freiburg, Hermann-Herder-Str. 3, 79104 Freiburg, Germany.

[†]These authors contributed equally

*Correspondence to: awtsen@uwaterloo.ca

Supplementary Note 1: Photocurrent Characteristics

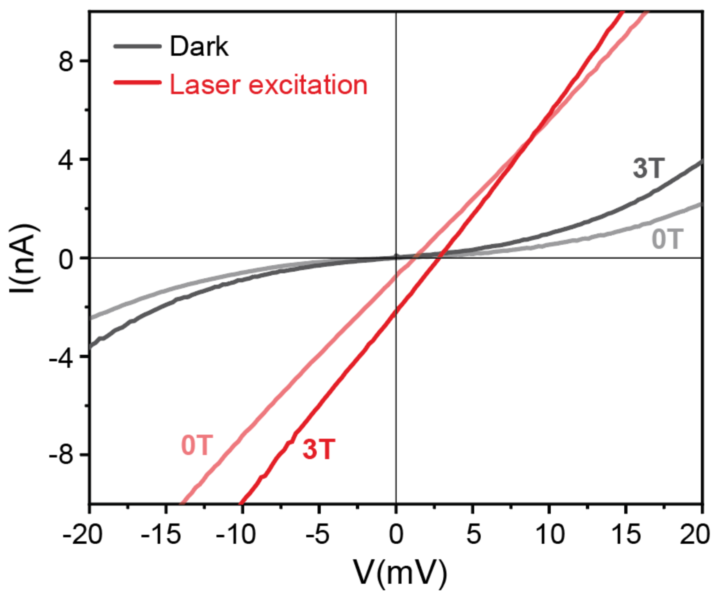

Supplementary Figure 1 | Current-voltage characteristics of 1.05 degree tDB CrI_3 device shown in the main text (Device 1) with and without laser illumination for two out-of-plane magnetic field levels. All I_{pc} measurements shown in the main text were performed under zero-bias conditions.

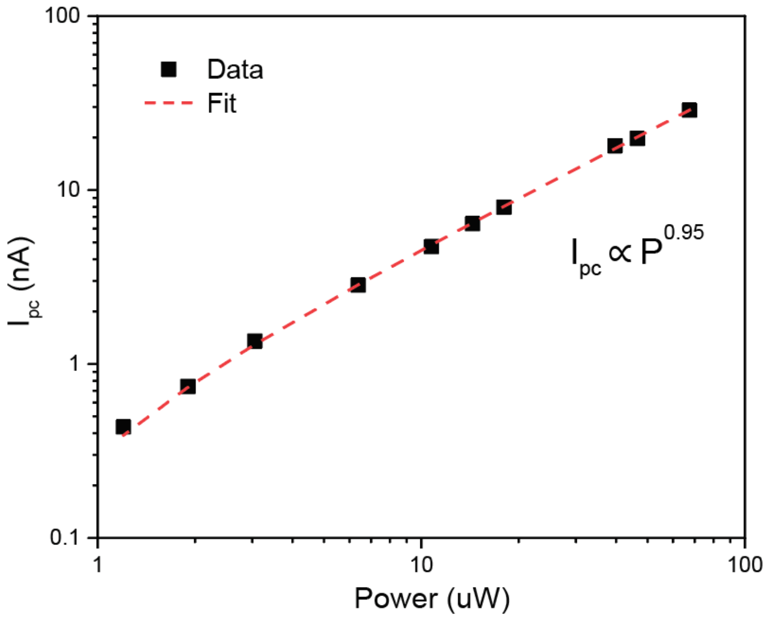

Supplementary Figure 2 | I_{pc} laser power dependence for Device 1. A power law fit yields an exponent of 0.95, nearly linear across the entire range. For all I_{pc} measurements taken at fixed laser power, ~10µW was used.

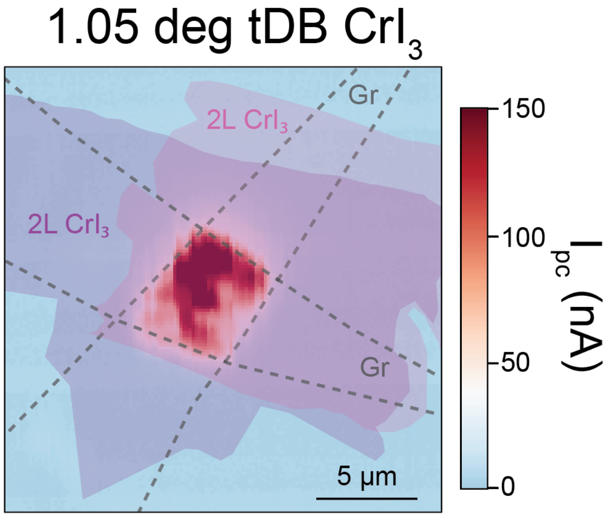

Supplementary Figure 3 | Spatial imaging of I_{pc} response. Photocurrent imaging shows a high I_{pc} response from the overlap region of the two Gr electrodes with the tDB CrI_3. The entire area is encapsulated in hBN.

Supplementary Note 2: Photocurrent and Reflection MCD Measurements

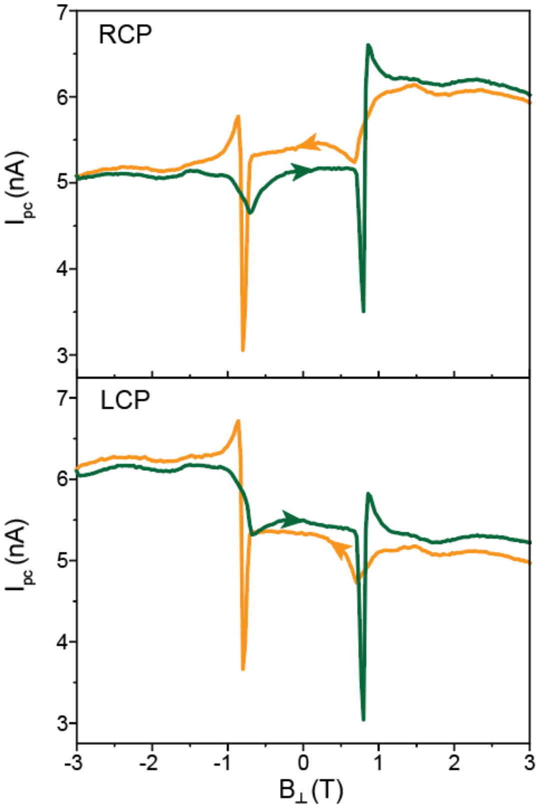

Supplementary Figure 4 | I_{pc} vs out-of-plane magnetic field under circularly polarized light for Device 1. The asymmetric transitions at ±0.8T observed in I_{pc}-MCD (RCP - LCP) and TMR are also seen in the single-channel I_{pc} data. For a given sweep direction, one transition shows a single peak, while a double-peak structure is seen for the second in both the RCP and LCP channels.

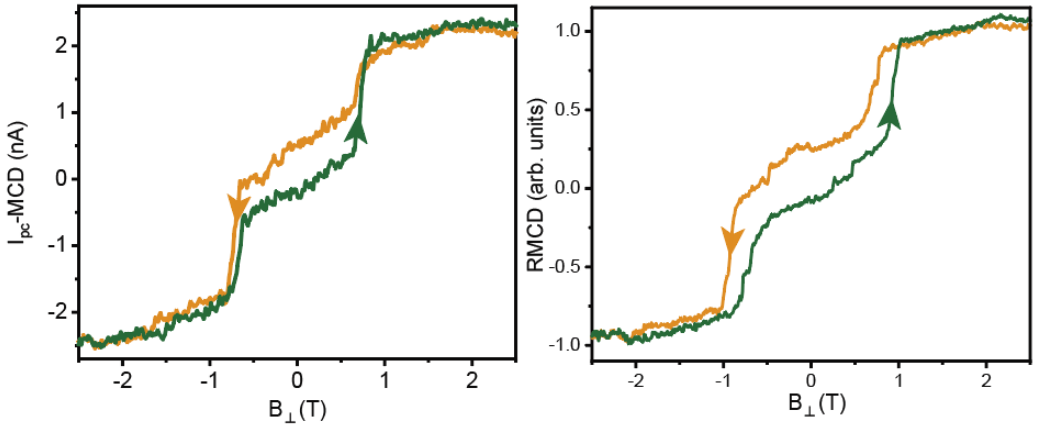

Supplementary Figure 5 | I_{pc}-MCD and reflection MCD taken on second 1.05 degree tDB CrI_3 device (Device 2). The remnant magnetization, slope, and FM loop around zero field are seen in both MCD measurements.

Supplementary Note 3: Determination of Interlayer Coupling in Twisted CrI_3

The interlayer coupling plays an important role in determining the local spin textures, since it competes with the exchange anisotropy, the intralayer coupling, and the external magnetic field. Here we discuss how the interlayer coupling between the inner layers is obtained from the data shown in ref.[1], and clarify the subtle issues involved in fitting the data.

Since the moiré lattice is periodic, the interlayer coupling $J(\boldsymbol{R})$ must respect this periodicity. Therefore, in order to extract the interlayer coupling from Fig. 2 and 3 in ref.[1], we express it in a Fourier decomposed form,

$$J(\boldsymbol{R}) = \sum_{n,m} C_{nm} \cos[(n\,\boldsymbol{G}_1 + m\,\boldsymbol{G}_2) \cdot \boldsymbol{R} + \phi_{nm}],$$

where $\boldsymbol{G}_1 = 2\pi L^{-1}\left(1, -\frac{1}{\sqrt{3}}\right)$ and $\boldsymbol{G}_2 = 2\pi L^{-1}\left(0, \frac{2}{\sqrt{3}}\right)$ are the reciprocal vectors of the moiré lattice spanned by the primitive vectors $\boldsymbol{a}_1 = L(1,0)$ and $\boldsymbol{a}_2 = L\left(\frac{1}{2}, \frac{\sqrt{3}}{2}\right)$. It is clear that $J(\boldsymbol{R})$ is invariant under the translation by the primitive vectors. Due to the C_3-symmetry, the Fourier coefficients must satisfy $C_{nm} = C_{m-n,-n} = C_{-m,n-m}$ and $\phi_{nm} = \phi_{m-n,-n} = \phi_{-m,n-m}$.

From ref.[1], the estimated values of $J(\boldsymbol{R})$ for $\boldsymbol{R}$ on the diagonal and edges of one moiré unit cell can be obtained, $\{J'(\boldsymbol{R}_i)\}$. We then use the least squares fitting to obtain the best fitting parameters C_{nm} and ϕ_{nm}. We found that including $(n, m) = (1,0), (1, -1), (2, 0)$ and $(2, -1)$ (and the combinations related by the symmetries) is sufficient to reproduce Fig. 1b in the main text. Including more fitting parameters leads to overfitting and the results become too sensitive to the initial fitting estimates.

Supplementary Note 4: Field-dependent Simulations of Moiré Spin Textures

Hamiltonian

The tDB CrI_3 system consists of four layers of honeycomb lattices. The Hamiltonian of each layer H^l is given by a generalized Heisenberg model with exchange anisotropy and Zeeman interaction:

$$H^l = -\sum_{\langle i,j\rangle}(J_0\, \boldsymbol{M}_{i,l} \cdot \boldsymbol{M}_{j,l} + K_0 M_{i,l}^z M_{j,l}^z) - g\mu_B \sum_i \boldsymbol{M}_{i,l} \cdot \boldsymbol{B},$$

where l is the layer index and i, j are the site indices. Each spin $\boldsymbol{M}_{i,l}$ is a classical three-dimensional vector of length $3/2$. The first two terms represent the intralayer exchange coupling J_0 and the exchange anisotropy K_0 between nearest-neighbor sites $\langle i,j\rangle$. We set $J_0 = 2.25$ and $K_0 = 0.09$ measured in meV[2]. The second term is the Zeeman term with g-factor ~ 2.1788[2].

The spins in adjacent layers are coupled by interlayer exchange coupling that is obtained from ref.[1]. The interlayer coupling is given by:

$$H^{\mathrm{int}} = -\textstyle\sum_{l=1}^{3} \sum_i J_i^{l,l+1}\, \boldsymbol{M}_{i,l} \cdot \boldsymbol{M}_{i,l+1}.$$

Only the coupling between the second and the third layer $J^{2,3}$ is spatially modulated. The other two interlayer couplings are spatially homogeneous and antiferromagnetic, $J^{1,2} = J^{3,4} = J_0^{\perp} < 0$. Supplementary Figure 6 shows the simulated spin structure arising from the effective interlayer coupling without any applied magnetic field.

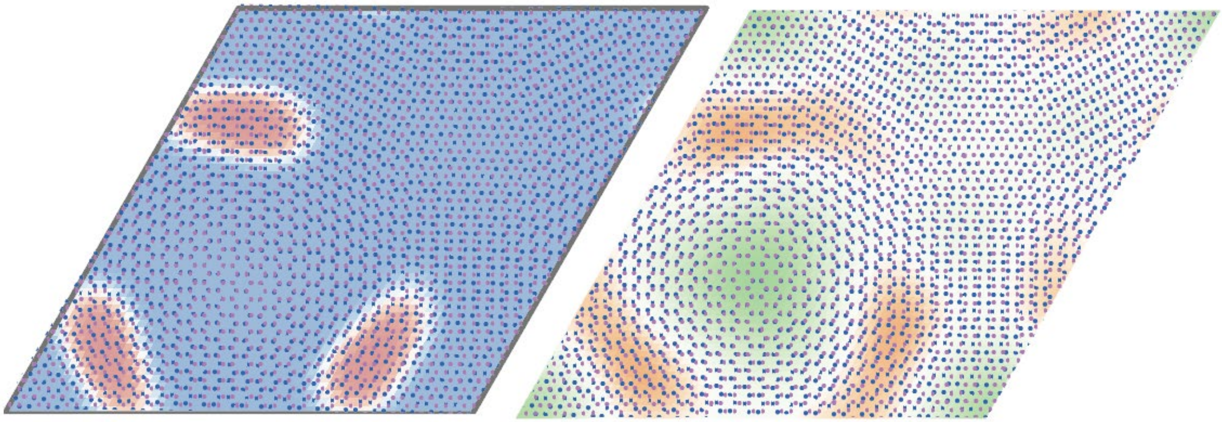

Supplementary Figure 6 | Comparison between simulated spin structure and spatially dependent interlayer coupling. The simulated spin structure (left) is taken from layer 3 in Fig. 2b (II). $J_{\perp}(\boldsymbol{r})$ (right) is from Fig. 1c. The lattice structure is overlaid for both. Comparison between the two reveals that the skyrmion domains are formed at the regions with strongest AFM interlayer coupling, i.e., AB_1' and AC stacking sites.

Each simulation run (i.e., magnetic field sweep in a single direction) starts with randomly initialized spins. In the presence of the initial magnetic field, we minimize the total energy by the semi-implicit method combined with backtracking line search [see Chap. 3 of ref.[3]]. The method corresponds to spin updates based on the damping term of the Landau-Lifshitz-Gilbert equation with optimal time steps. When the

minimization is finished, we record the spin configuration and move on to the next value of the magnetic fields. Before minimizing the energy at the new magnetic field, we add stochastic randomness sampled from a Gaussian distribution to the spins in order to prevent the system from being trapped in a local minimum. The width of the hysteresis loop (for example, see Fig. 2a) depends on the amount of randomness added between each step. In Supplementary Figure 7 we show 3 different runs with the same model used in Fig. 2 but with varying randomness. For randomness with higher magnitude, the sharp transition from the fully polarized-up state to the ground state near 0T is in the range of 0.5T-1T. For lower randomness, the same transition is delayed until the ground state is much more energetically favoured, very close to 0T. For Fig. 2 we set the randomness to be 50, for which the transition fields are closer to experimental results.

We note that with higher randomness out-of-plane domains are more likely to transfer from one layer to another (no transfer of out-of-plane domains is observed for the R=5 case) between adjacent steps. Such a transfer is not a physical process that should occur within a moiré unit cell since the energy barrier between the two configurations is reasonably high. Instead, as in classical Monte Carlo simulations, it merely indicates that these configurations have similar energies and are sampled equally by the randomness.

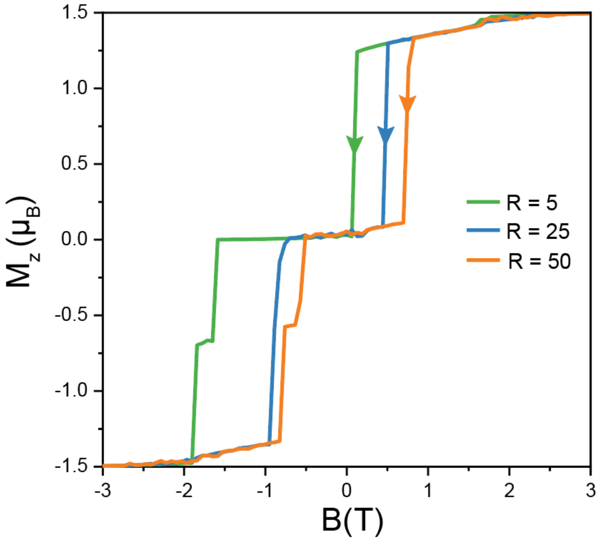

Supplementary Figure 7 | Effect of randomness on micromagnetic simulations. Between each field step, randomness is added to the magnetic state to remove the system from any local minimum. Traces are shown for three sweeps with increasing amount of randomness added to the system between each step. For higher randomness, the transition field value matches better with experimental data.

Selection between rigid and relaxed models

The interlayer coupling depends critically on whether the atomic positions are relaxed in the density-functional calculations[1]. Without relaxation, the interlayer coupling is five to ten times more antiferromagnetic at the AB_1' and AC' stacking sites as shown in Supplementary Figure 8. When we use such interlayer coupling, the simulations do not agree with several experimental features. In Fig. Supplementary Figure 9a we show the simulated out-of-plane component of magnetization (M_z) using the parameters for the unrelaxed or the rigid model. The curve has a much smaller remnant magnetization or hysteresis loop compared to that for the relaxed model shown in Fig. 2. Spin structures shown in Supplementary Figure 9b indicate that this is due to the formation of domains on outer layers that are AFM

coupled to the domains on the inner layers. We also observe that all the spins are not completely polarized with an out-of-plane field of 3T.

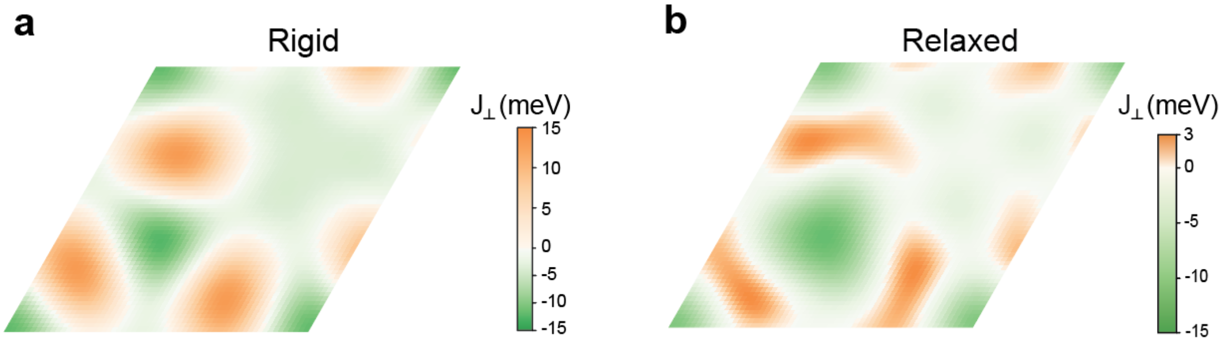

Supplementary Figure 8 | Comparison of spatially modulated interlayer coupling between rigid and relaxed models. (a): Interlayer coupling calculated for the rigid or unrelaxed crystal lattice across the moiré unit cell. (b): After relaxing the lattice, the AFM coupling energy in the orange regions are substantially reduced. Note the different scale bars.

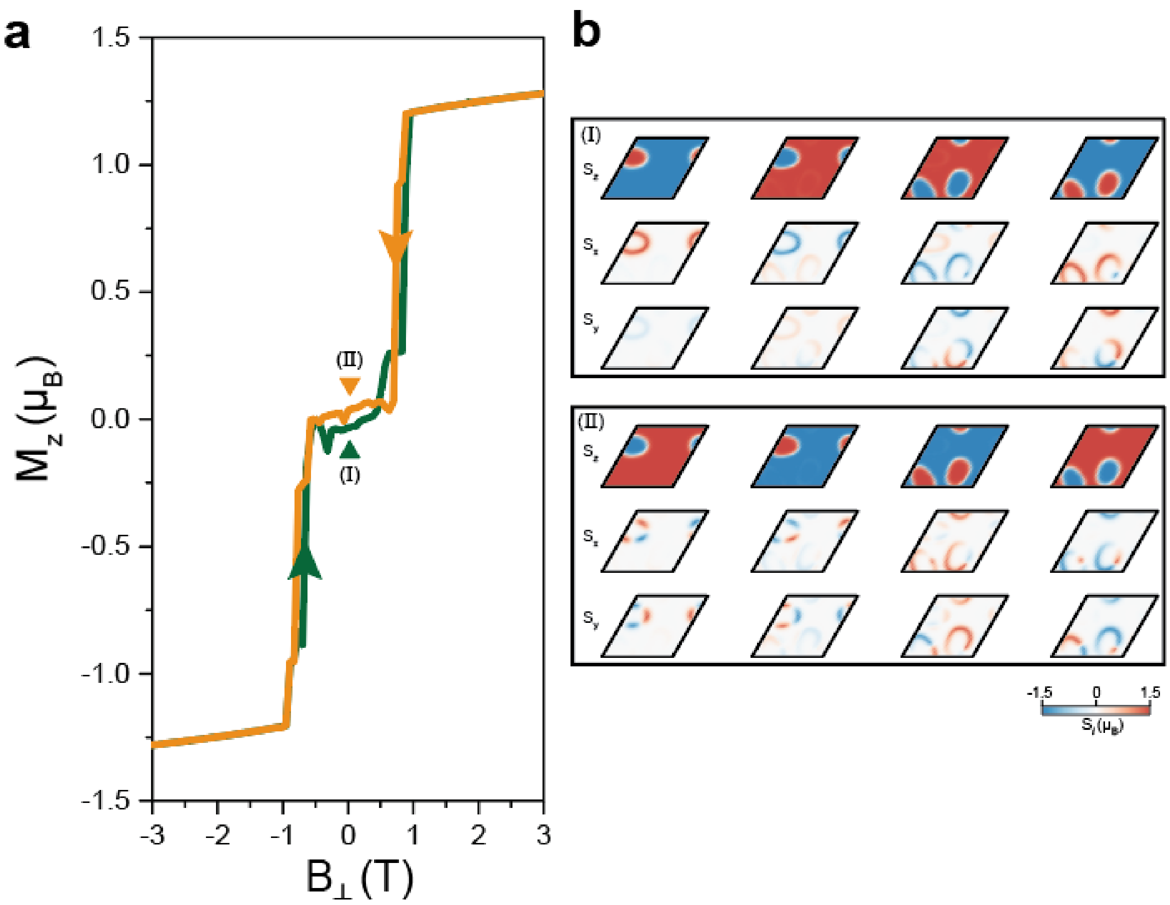

Supplementary Figure 9 | Simulation of magnetization and spin textures with interlayer coupling from a rigid lattice. (a): Net out-of-plane magnetization as a function of out-of-plane magnetic field for both sweep directions -3T to +3T (green trace) and +3T to -3T (orange trace). (b): S_x, S_y, and S_z spin structures for all the four layers at 0T for two opposing ground states (marked by arrows in (a)).

We next show how TMR under an in-plane magnetic field can be used to check the validity of the two models. In Supplementary Figure 10a we plot the numerically calculated TMR for an applied in-plane magnetic field with interlayer coupling of the rigid (black) and relaxed (red) model (see Supplementary Note 6 for details of calculation). We overlay the measured in plane TMR (blue-dashed) over these traces. It can be observed that the measured TMR saturates at around 7T. The calculated transmission for the relaxed model saturates at around 8T, but for the rigid model it does not saturate even up to 15T. This is corroborated by the spin structures shown in Supplementary Figure 10b. Thus, throughout this work, we have used the interlayer coupling obtained from either the fully relaxed or dominantly relaxed model (see section below).

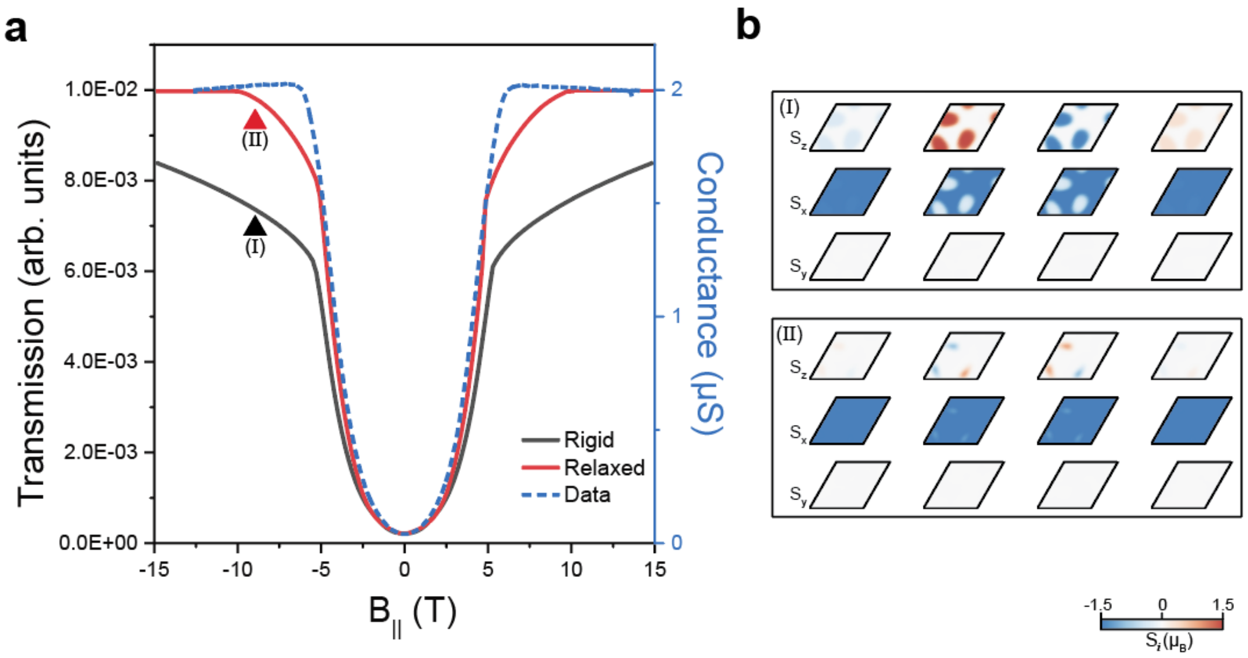

Supplementary Figure 10 | Simulated tunneling transmission vs in-plane magnetic field for rigid and relaxed interlayer coupling. (a): Numerically calculated tunneling probability for tDB CrI_3 with interlayer coupling from relaxed (red) and rigid (black) models as a function of applied in-plane magnetic field. The experimental data is overlaid in dashed blue. The tunneling for rigid model does not saturate up to 15T (b): Simulated spin structures (at field level marked by arrows in (a)) for the two models show that spins in the relaxed model (II) are nearly fully polarized at ~8T, while that for the rigid model (I) contain substantial spins still pointing out-of-plane.

Linear interpolation between the relaxed and rigid models

In Supplementary Figure 11a, we show the simulated M_z curve from a system with 90% relaxed and 10% rigid interlayer coupling to highlight the sensitivity of the spin model to the set interlayer coupling. The spin structure at 0T (Supplementary Figure 11b) is still qualitatively similar to that for the relaxed system, but with slightly larger domains. This is reflected in a larger hysteresis loop or remnant magnetization. While the model can be more finely tuned to match the experimental data, the salient features are already captured in the fully relaxed model.

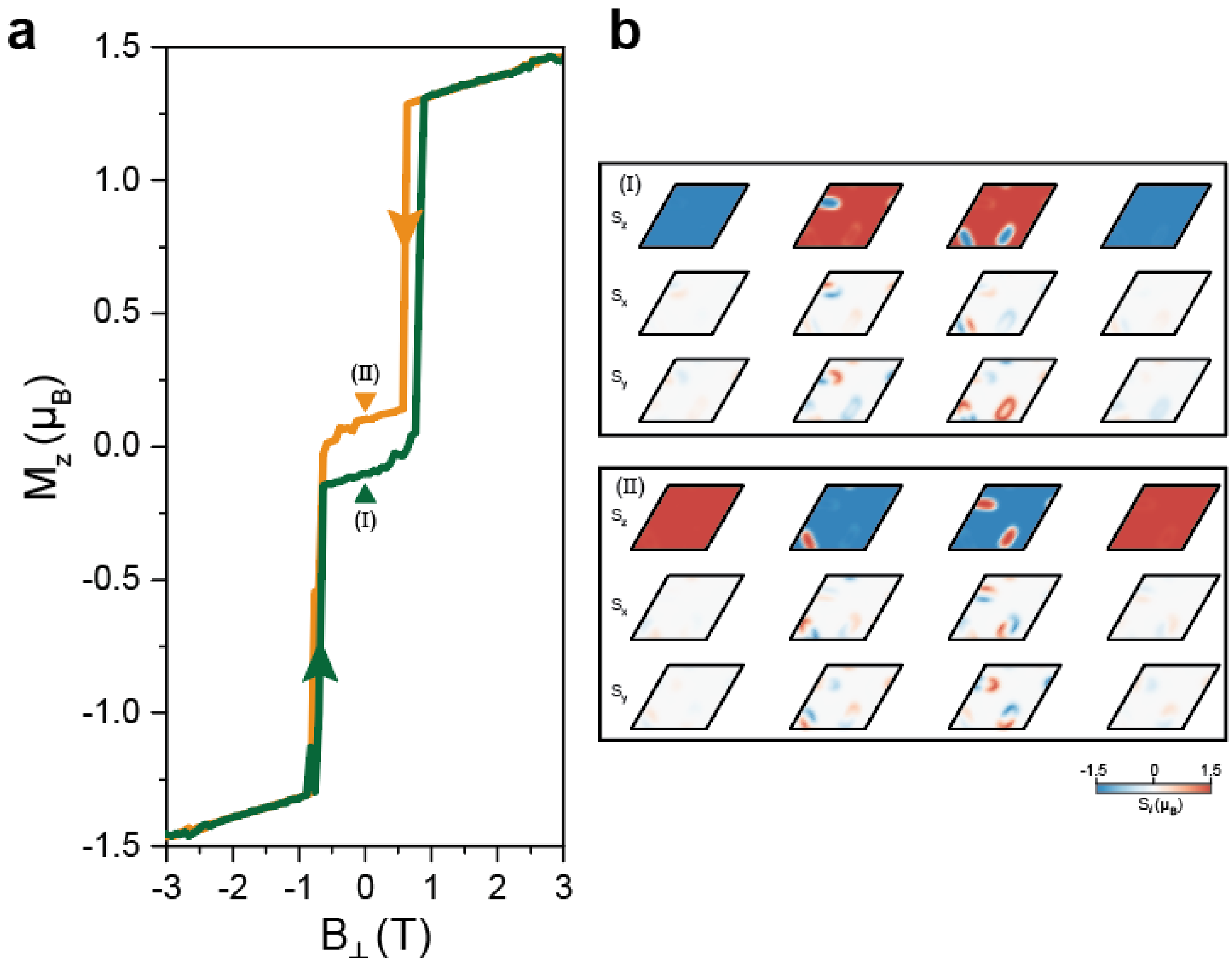

Supplementary Figure 11 | A mixed model of interlayer coupling. (a): Simulated magnetization curve using interlayer coupling from 90% relaxed and 10% rigid model as a function of out-of-plane magnetic field. (b): Ground state spin structures at 0T show that magnetic domains in the inner layers are slightly larger than those from the fully relaxed model. No domains are seen on the outer layers.

Supplementary Note 5: Memory-dependent Device Response

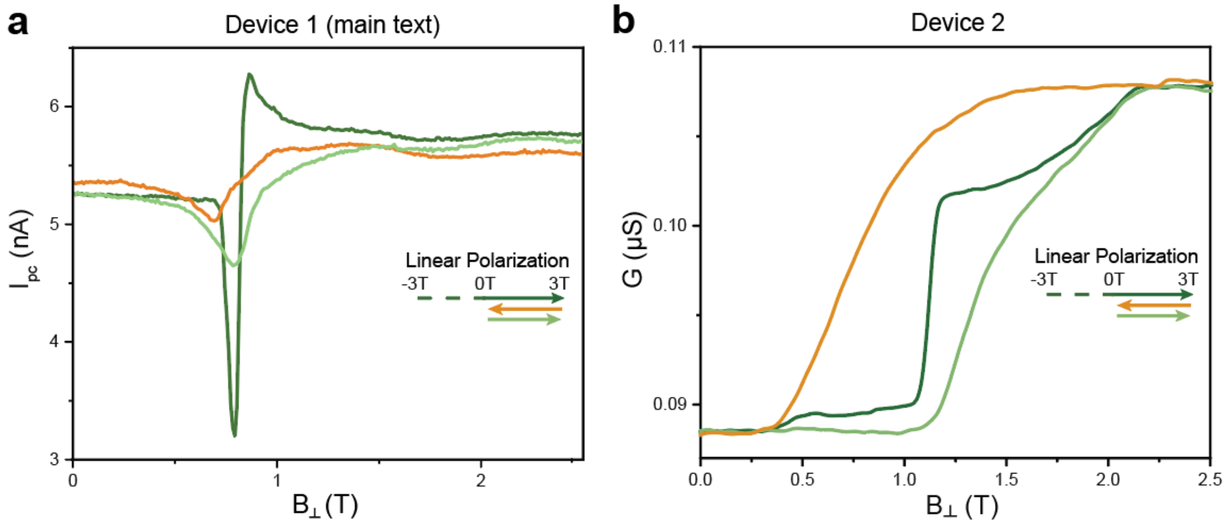

Supplementary Figure 12 | Memory-dependent I_{pc} and tunneling magnetoconductance under out-of-plane magnetic field. (a): When various magnetic field sweep conditions used in the TMR measurements in Fig. 3 are applied for photocurrent (Device 1), a similar memory-dependent effect is observed for the transition at ~0.8T. The different jump characteristics are also consistent with those seen at ±0.8T in Supplementary Figure 4. (b): Memory-dependent TMR behavior in Fig. 3 is reproduced in Device 2. The gradual change between 1T and 2T for the dark green trace is even more apparent.

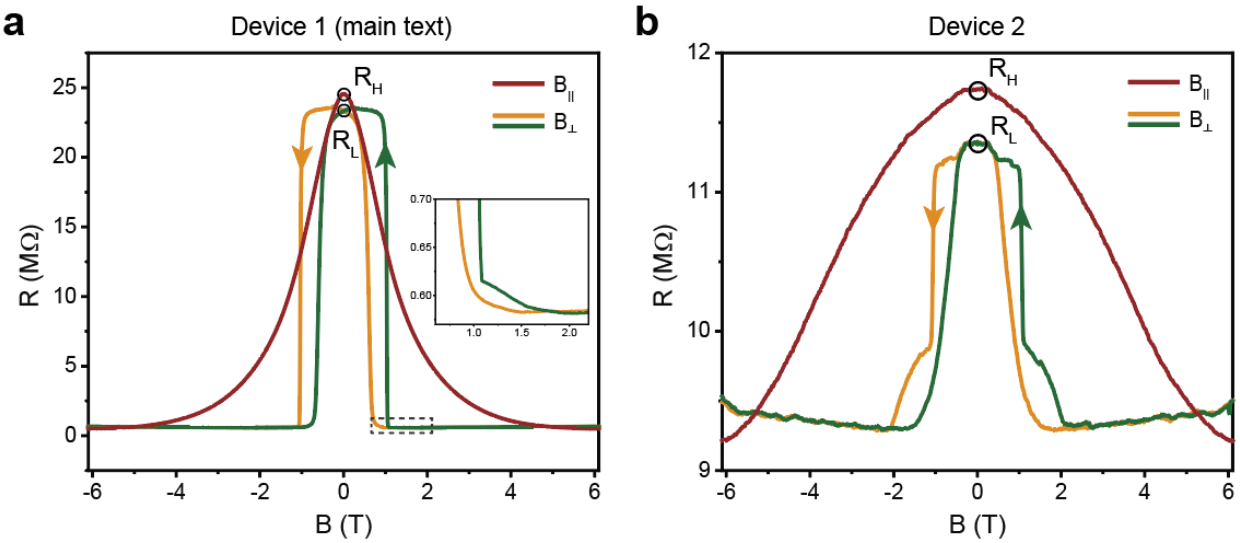

Supplementary Figure 13 | TMR of 1.05 degree tDB CrI_3 devices between -6T and +6T. (a): The TMR data in Fig. 4a is shown for a larger field range. The inset shows a zoom-in of the out-of-plane data around the transition at ~1T. (b): Similar TMR data taken on Device 2. For both devices, the two transitions at ~ ±1T for out-of-plane field are asymmetric, consistent with photocurrent data. Two different resistance states at zero field (R_H and R_L) are also seen for both. For Device 1 (2), R_L state is ~6% (~3.4%) lower than R_H.

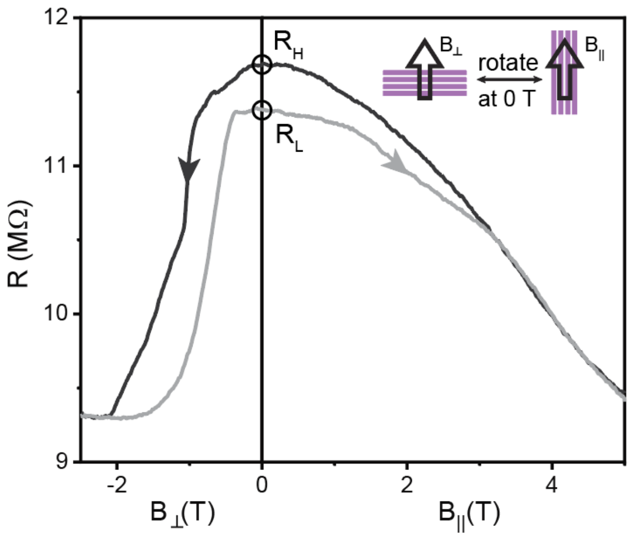

Supplementary Figure 14 | Reversible switching between R_H and R_L is reproduced in Device 2.

Supplementary Note 6: Tunneling Transmission Calculation

Generalized Transmission Formula

In this note, we derive the generalized transmission rate for unpolarized electrons tunneling through four CrI_3 layers with arbitrary spin orientations, as shown in Supplementary Figure 15a. We assume that the tunneling path is perpendicular to the lattice planes and no lateral conduction channel is considered. Without loss of generality, the initial unpolarized input state can be written as,

$$\hat{\rho}_0 = \frac{1}{2}\,|\uparrow\rangle_1\langle\uparrow|_1 \;+ \frac{1}{2}\,|\downarrow\rangle_1\langle\downarrow|_1, \qquad [1]$$

where $\{\,|\uparrow\rangle_k\,, |\downarrow\rangle_k\}$ are the basis vectors in layer k.

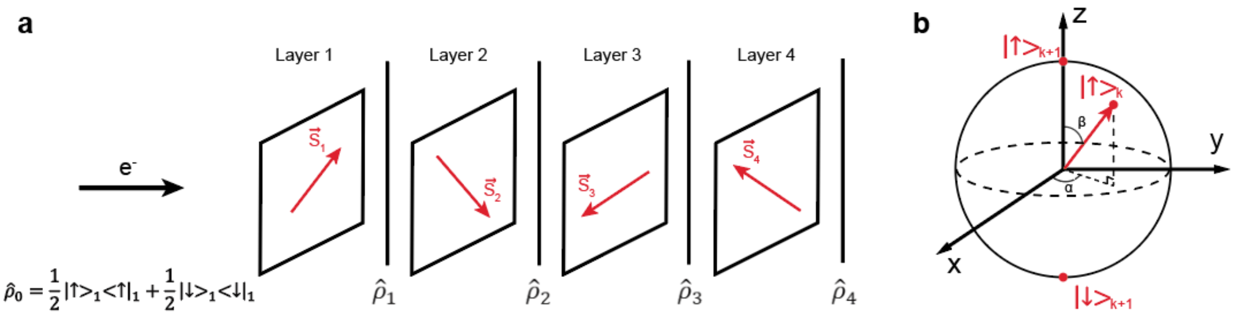

Supplementary Figure 15 | Derivation of transmission formula for untwisted and tDB CrI_3. (a): Schematic picture used for deriving the transmission formula. The density matrix of electrons before and after each layer is denoted as $\hat{\rho}_i$, where $i = 0,1,2,3$. Spins on each layer, $\boldsymbol{S_i}$, can point in any arbitrary direction. (b): The relative angles, α and β, between two adjacent spin states $(|\uparrow\rangle_k, |\uparrow\rangle_{k+1})$ are defined on the Bloch sphere.

After layer 1, the density matrix is modified by the transmission rates:

$$\hat{\rho}_1 = \frac{T_\uparrow}{2}\,|\uparrow\rangle_1\langle\uparrow|_1 \;+ \frac{T_\downarrow}{2}\,|\downarrow\rangle_1\langle\downarrow|_1 = (|\uparrow\rangle_1 \quad |\downarrow\rangle_1)\begin{pmatrix} \frac{T_\uparrow}{2} & 0 \\ 0 & \frac{T_\downarrow}{2} \end{pmatrix}\begin{pmatrix} \langle\uparrow|_1 \\ \langle\downarrow|_1 \end{pmatrix}, \qquad [2]$$

where $T_\uparrow$ and $T_\downarrow$ denote the transmission coefficients for tunneling spins parallel and antiparallel to the local spins, $\boldsymbol{S_k}$, respectively. We only focus on the transmitted components, and ignore the reflected components (Thus, the trace of $\hat{\rho}_1$ is smaller than 1).

The density matrix should be spanned by the new set of bases $\{\,|\uparrow\rangle_2\,, |\downarrow\rangle_2\}$ in layer 2, with a transform matrix, U_{12}, given by,

$$U_{12} = \begin{pmatrix} \cos\frac{\beta_{12}}{2}\exp\left(\frac{i\alpha_{12}}{2}\right) & \sin\frac{\beta_{12}}{2}\exp\left(-\frac{i\alpha_{12}}{2}\right) \\ -\sin\frac{\beta_{12}}{2}\exp\left(\frac{i\alpha_{12}}{2}\right) & \cos\frac{\beta_{12}}{2}\exp\left(-\frac{i\alpha_{12}}{2}\right) \end{pmatrix}, \qquad [3]$$

where the respective angles, α and β, are labeled on a Bloch sphere in Supplementary Figure 15b. In the new basis, the density matrix is now written as

$$\hat{\rho}_1 = (|\uparrow\rangle_2 \quad |\downarrow\rangle_2) U_{12}^T \begin{pmatrix} \frac{T_\uparrow}{2} & 0 \\ 0 & \frac{T_\downarrow}{2} \end{pmatrix} U_{12}^* \begin{pmatrix} \langle\uparrow|_2 \\ \langle\downarrow|_2 \end{pmatrix}$$
$$\equiv (|\uparrow\rangle_2 \quad |\downarrow\rangle_2) \begin{pmatrix} \rho_{\uparrow\uparrow}^1 & \rho_{\uparrow\downarrow}^1 \\ \rho_{\downarrow\uparrow}^1 & \rho_{\downarrow\downarrow}^1 \end{pmatrix} \begin{pmatrix} \langle\uparrow|_2 \\ \langle\downarrow|_2 \end{pmatrix}. \quad [4]$$

where $\rho_{\uparrow\uparrow}^1 \equiv \frac{T_\uparrow}{2}\cos^2\frac{\beta_{12}}{2} + \frac{T_\downarrow}{2}\sin^2\frac{\beta_{12}}{2}$, and other terms are defined in an equivalent way. After layer 2, the density matrix is given as,

$$\hat{\rho}_2 = T_\uparrow\rho_{\uparrow\uparrow}^1 \,|\uparrow\rangle_2\langle\uparrow|_2 + T_\downarrow\rho_{\downarrow\downarrow}^1 \,|\downarrow\rangle_2\langle\downarrow|_2 = (|\uparrow\rangle_2 \quad |\downarrow\rangle_2) \begin{pmatrix} T_\uparrow\rho_{\uparrow\uparrow}^1 & 0 \\ 0 & T_\downarrow\rho_{\downarrow\downarrow}^1 \end{pmatrix} \begin{pmatrix} \langle\uparrow|_2 \\ \langle\downarrow|_2 \end{pmatrix}. \quad [5]$$

We ignore the coherent part of the density matrix assuming that no spin-flip processes occur while electrons pass through layer 2. By repeatedly applying the transformation and transmission coefficients to layer 3 and layer 4, the density matrix ends up as,

$$\hat{\rho}_4 = T_\uparrow\rho_{\uparrow\uparrow}^3 \,|\uparrow\rangle_4\langle\uparrow|_4 + T_\downarrow\rho_{\downarrow\downarrow}^3 \,|\downarrow\rangle_4\langle\downarrow|_4 \quad [6]$$

The total transmission rate is calculated to be,

$$
\begin{aligned}
T_{total} &= T_{\uparrow}\rho_{\uparrow\uparrow}^{3} + T_{\downarrow}\rho_{\downarrow\downarrow}^{3} \\
&= \left(\left(\frac{T_{\uparrow}^{4}}{2}\cos^2\frac{\beta_{12}}{2} + \frac{T_{\uparrow}^{3}T_{\downarrow}}{2}\sin^2\frac{\beta_{12}}{2}\right)\cos^2\frac{\beta_{23}}{2}\right. \\
&\quad \left. + \left(\frac{T_{\uparrow}^{3}T_{\downarrow}}{2}\sin^2\frac{\beta_{12}}{2} + \frac{T_{\uparrow}^{2}T_{\downarrow}^{2}}{2}\cos^2\frac{\beta_{12}}{2}\right)\sin^2\frac{\beta_{23}}{2}\right)\cos^2\frac{\beta_{34}}{2} \\
&\quad + \left(\left(\frac{T_{\uparrow}^{3}T_{\downarrow}}{2}\cos^2\frac{\beta_{12}}{2} + \frac{T_{\uparrow}^{2}T_{\downarrow}^{2}}{2}\sin^2\frac{\beta_{12}}{2}\right)\sin^2\frac{\beta_{23}}{2}\right. \\
&\quad \left. + \left(\frac{T_{\uparrow}^{2}T_{\downarrow}^{2}}{2}\sin^2\frac{\beta_{12}}{2} + \frac{T_{\uparrow}T_{\downarrow}^{3}}{2}\cos^2\frac{\beta_{12}}{2}\right)\cos^2\frac{\beta_{23}}{2}\right)\sin^2\frac{\beta_{34}}{2} \\
&\quad + \left(\left(\frac{T_{\uparrow}^{3}T_{\downarrow}}{2}\cos^2\frac{\beta_{12}}{2} + \frac{T_{\uparrow}^{2}T_{\downarrow}^{2}}{2}\sin^2\frac{\beta_{12}}{2}\right)\cos^2\frac{\beta_{23}}{2}\right. \\
&\quad \left. + \left(\frac{T_{\uparrow}^{2}T_{\downarrow}^{2}}{2}\sin^2\frac{\beta_{12}}{2} + \frac{T_{\uparrow}T_{\downarrow}^{3}}{2}\cos^2\frac{\beta_{12}}{2}\right)\sin^2\frac{\beta_{23}}{2}\right)\sin^2\frac{\beta_{34}}{2} \\
&\quad + \left(\left(\frac{T_{\uparrow}^{2}T_{\downarrow}^{2}}{2}\cos^2\frac{\beta_{12}}{2} + \frac{T_{\uparrow}T_{\downarrow}^{3}}{2}\sin^2\frac{\beta_{12}}{2}\right)\sin^2\frac{\beta_{23}}{2}\right. \\
&\quad \left. + \left(\frac{T_{\uparrow}T_{\downarrow}^{3}}{2}\sin^2\frac{\beta_{12}}{2} + \frac{T_{\downarrow}^{4}}{2}\cos^2\frac{\beta_{12}}{2}\right)\cos^2\frac{\beta_{23}}{2}\right)\cos^2\frac{\beta_{34}}{2}
\end{aligned} \qquad [7]
$$

Based on the derived transmission formula, the ratio of parallel (all $\beta_{k,k+1}$ set to be 0°) and antiparallel (all $\beta_{k,k+1}$ set to be 180°) configurations for an untwisted 4L CrI_3 is

$$
\frac{T_P}{T_{AP}} = \frac{T_{\uparrow}^{4} + T_{\downarrow}^{4}}{2T_{\uparrow}^{2}T_{\downarrow}^{2}} = \frac{r^4 + 1}{2r^2} \qquad [8]
$$

where $r = T_{\uparrow}/T_{\downarrow}$. This formula (Supplementary Equation 8) is consistent with that obtained in ref.[4]. With the ratio r, we can calculate the resistance ratios for the all the simulated spin textures.

Determination of Transmission Ratio

To determine the transmission coefficient ratio, r, we fabricate an untwisted device with an area of ~20μm². The large area allows us to measure conductance at zero DC bias. Supplementary Figure 16shows that at 0T and 2.1T (fully polarized state), the resistances are 5.5MΩ and 0.11MΩ, yielding a ratio of 50. Assigning the value to Supplementary Equation 8, we have the value of r equal to 10.

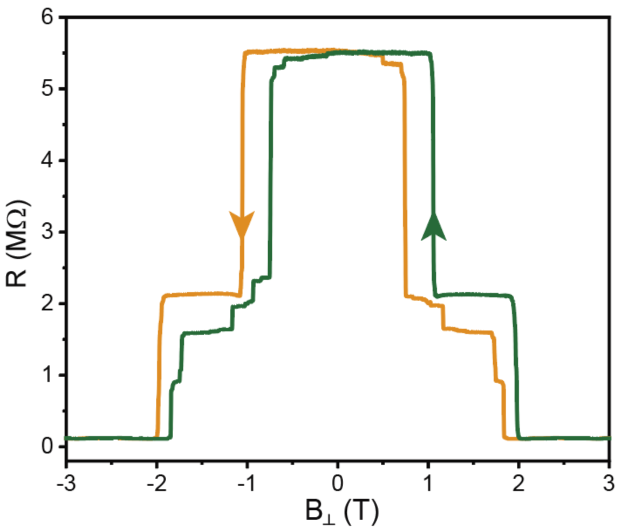

Supplementary Figure 16 | TMR of an untwisted four-layer CrI_3 with similar junction area as the tDB CrI_3 device shown in main text.

Analytical Analysis for the Two Types of Magnetic Domains

In this subsection, we apply the formulation form Supplementary Equation 1-7 to the center spins for the two proposed ground states in Fig. 1d, reproduced in Supplementary Figure 17below.

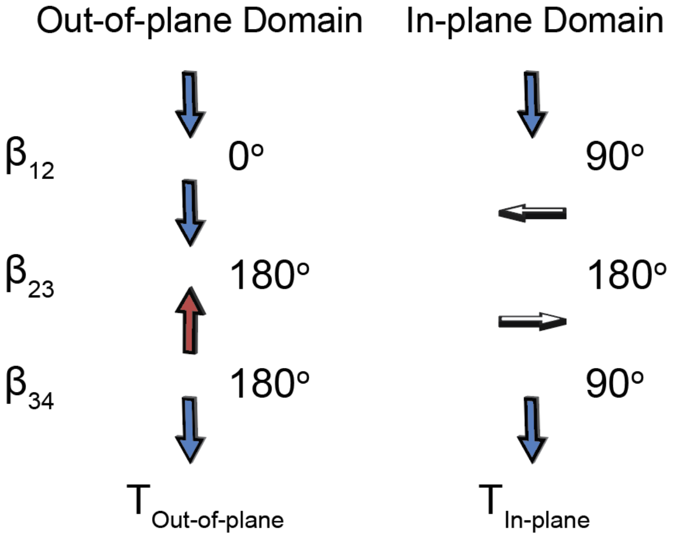

Supplementary Figure 17 | The center spins of an out-of-plane and in-plane domain on four atomic layers. The relative polar angles are labeled.

Substituting the values of β in the transform matrices, the transmission rates for the two cases are calculated to be,

$$T_{\text{out-of-plane}} = \frac{1}{2} T_{\uparrow}^{3} T_{\downarrow} + \frac{1}{2} T_{\downarrow}^{3} T_{\uparrow},$$

$$T_{in-plane} = \frac{1}{2}T_\uparrow^2 T_\downarrow^2 + \frac{1}{4}T_\uparrow^3 T_\downarrow + \frac{1}{4}T_\downarrow^3 T_\uparrow = T_{\text{out-of-plane}} - \frac{1}{4}T_\uparrow T_\downarrow (T_\uparrow - T_\downarrow)^2.$$

The second term of $T_{in-plane}$ is always negative, and thus the total transmission for a out-of-plane configuration is always larger (smaller resistance) than the one of in-plane domain configuration, which is consistent with the experimental results in Fig. 4a.

Numerical Calculation for the Two Types of Magnetic Domains

Out-of-plane domains usually constitutes either a magnetic skyrmion or a topologically trivial bubble depending on the winding of the in-plane spins along the domain wall, which can be quantified by winding $W = \frac{1}{4\pi}\int \mathbf{M}\cdot\left(\frac{\partial \mathbf{M}}{\partial x}\times\frac{\partial \mathbf{M}}{\partial y}\right)dxdy$, where $\mathbf{M}$ is the local magnetization[5,6]. Based on our micromagnetic simulation results, we can obtain all the relative polar angles β_{ij}, where i and j represent the two nearest CrI_3 layers. Using Supplementary Equation 7, we calculate the total transmission/resistance for the various spin structures.

Supplementary Figure 18a shows the total resistance of a complete moiré unit cell for the untwisted 4L CrI_3 and tDB CrI_3 in the R_H and R_L states. Here, the unitless resistance is defined as the reciprocal of total transmission, where $T_\uparrow$ and $T_\downarrow$ are set to be 0.316 and 0.0316 respectively, to satisfy the transmission ratio $r = 10$ (see Supplementary Equation 8). To obtain resistance of a single magnetic domain, the lattice is shifted by 5 lattice points along x- and y-directions to prevent clipping. Spins with z-projection >1.45 (<-1.45 if majority spins are negative) on the layer with the domain are masked out for all the 4 layers (see Supplementary Figure 18b). Left-bottom domains are selected in each case: for three skyrmions with different winding numbers and an in-plane domain. The resistance ratios mentioned in the main text (~13%) and the percentage difference between the two ground states shown in Fig. 4c are calculated by the resistance values shown in Supplementary Figure 18. We have also evaluated the energy for the two zero-field spin structures (out-of-plane and in-plane domain states). They are very close, making it possible to realize two metastable states at zero field.

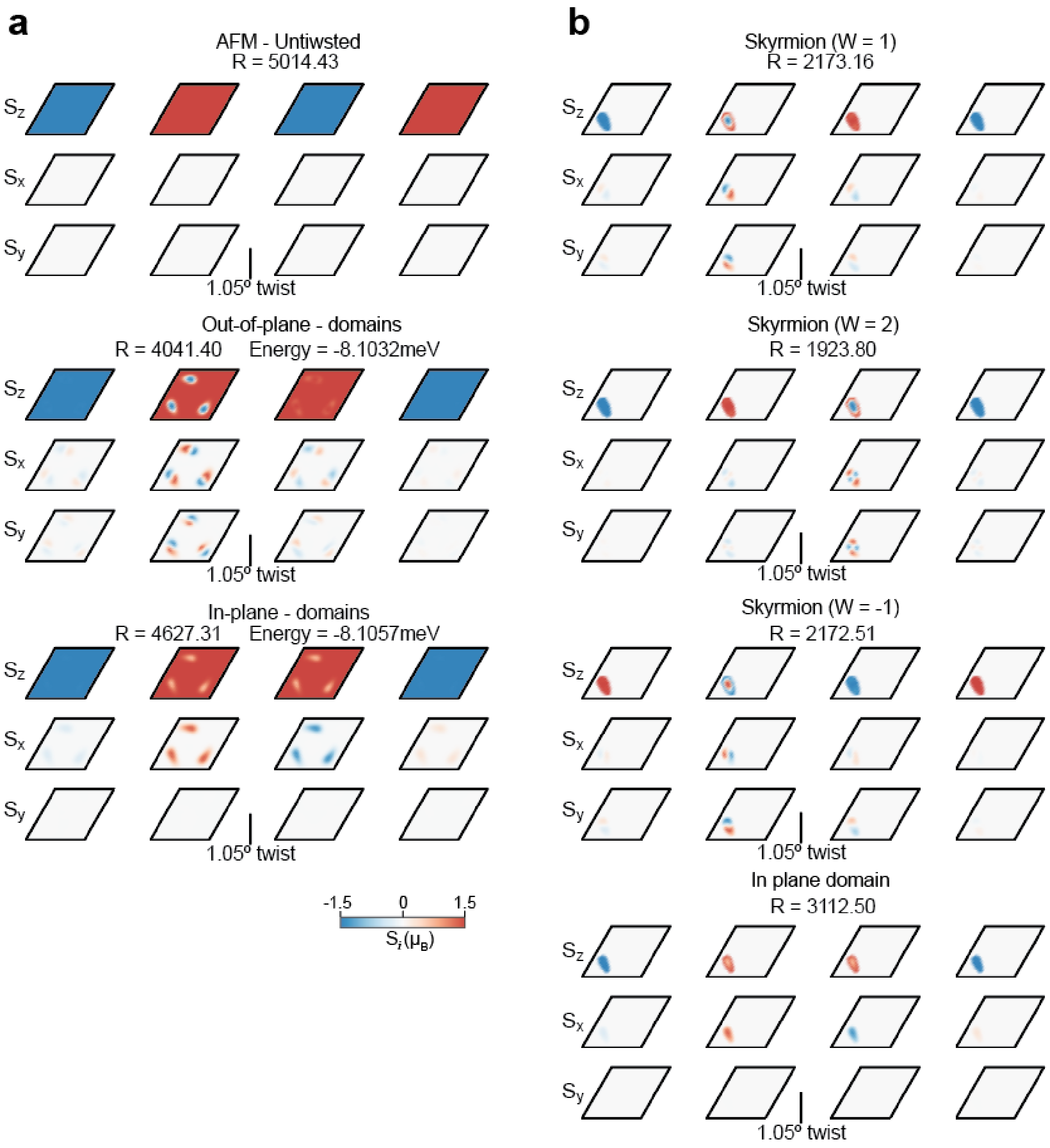

Supplementary Figure 18 | Calculated resistance (unitless) of moiré unit cells and single magnetic domains. (a): Moiré unit cells for untwisted four-layer CrI_3, and two ground state textures of tDB CrI_3. (b): Selected domains from skyrmion and in-plane domain states.

Supplementary Note 7: Twist Angle Dependence

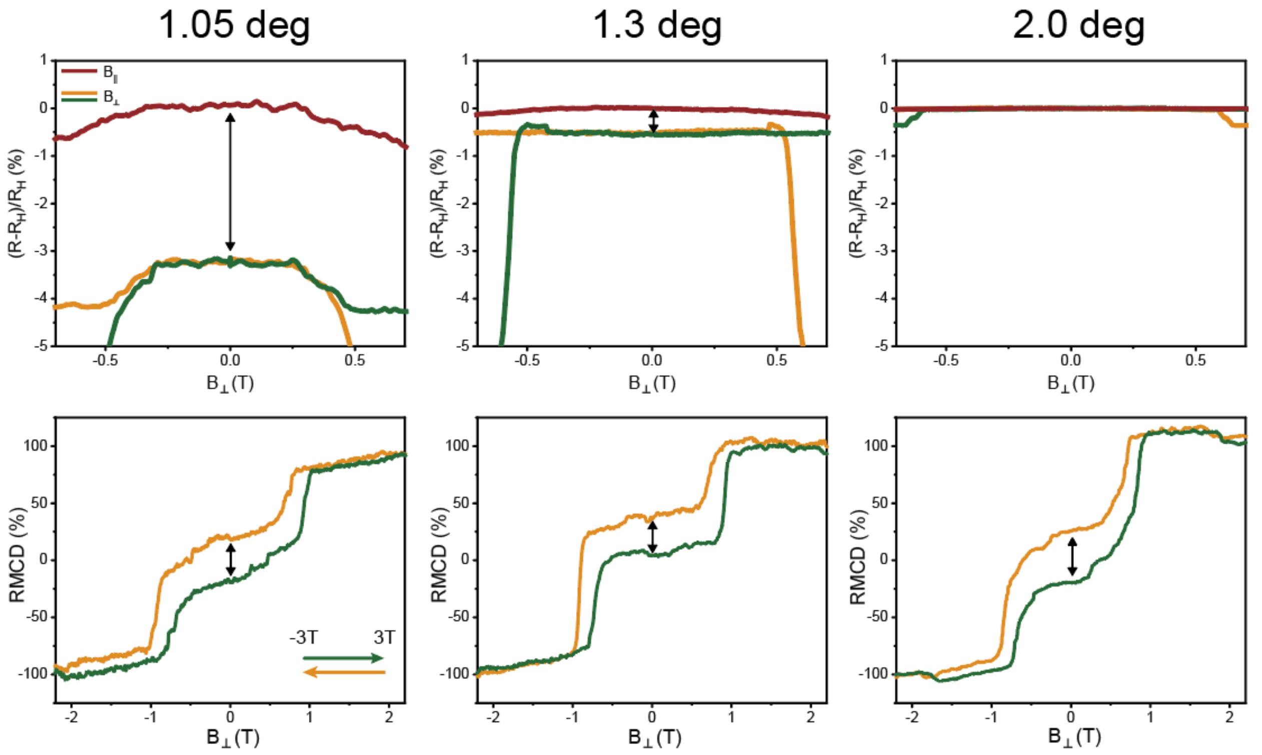

Supplementary Figure 19 | Comparison of TMR and reflection MCD taken for tDB CrI_3 devices with different twist angles: 1.05 degrees (Device 2), 1.3 degrees, and 2.0 degrees.

Twist angle dependent spin structure

Supplementary Figure 20 shows the results of simulations on tdB CrI_3 with twist angles of 1.3 degrees (left) and 2 degrees (right) with relaxed dominant interlayer coupling. The structure shown is at 0T after an applied magnetic field. For 1.3 degree twist angle, after an applied in-plane field, only in-plane domains are observed, while after an applied out-of-plane field, both in-plane and out-of-plane domains are observed. For 2 degree twist angle, only in-plane domains are observed at 0T after either an in-plane or out-of-plane magnetic field is applied. This is consistent with the experimental results from Fig. 5 and Supplementary Figure 19.

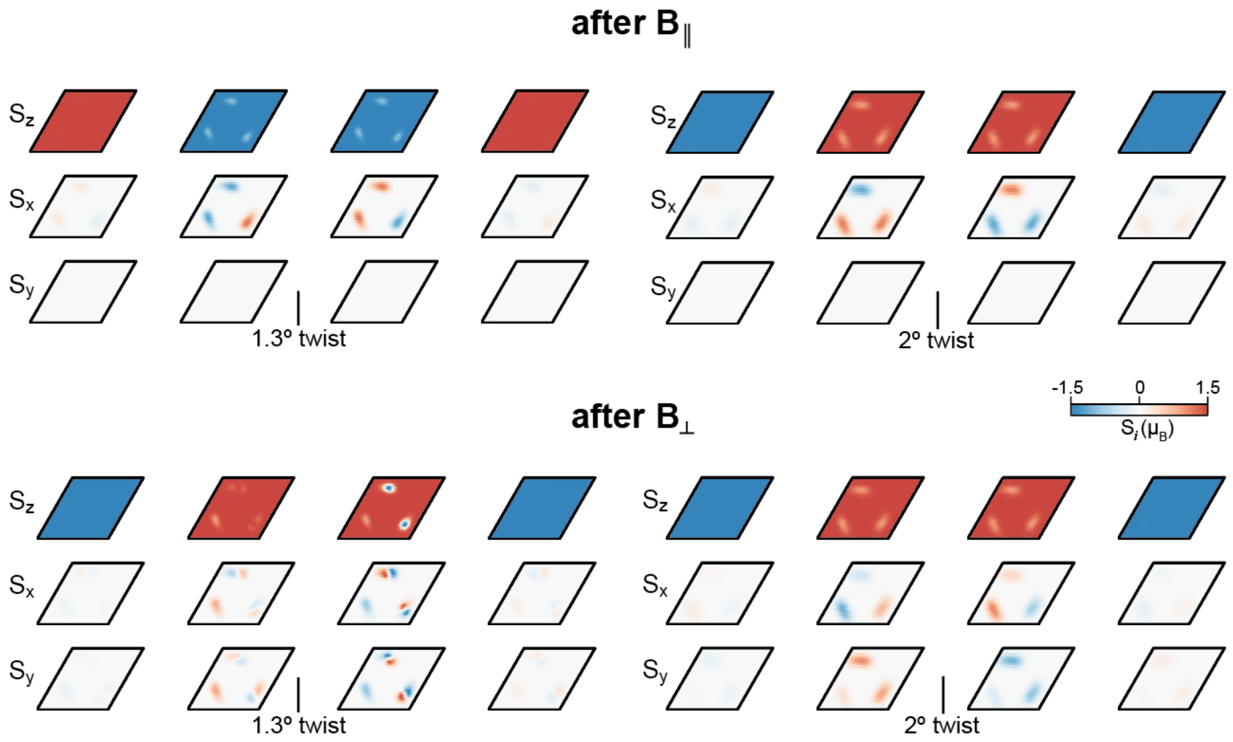

Supplementary Figure 20 | Simulated Sz, Sx and Sy spin structures of tDB CrI_3 with twist angle of 1.3 degrees (left) and 2 degrees (right) at 0T after an applied in-plane field (top) and out-of-plane field (bottom).

Supplementary References

1. Sivadas, N., Okamoto, S., Xu, X., Fennie, C. J. & Xiao, D. Stacking-Dependent Magnetism in Bilayer CrI_3. *Nano Lett.* **18**, 7658–7664 (2018).
2. Kim, H. H. *et al.* Evolution of interlayer and intralayer magnetism in three atomically thin chromium trihalides. *Proc. Natl. Acad. Sci. U. S. A.* **116**, 11131–11136 (2019).
3. Exl, L. Tensor grid methods for micromagnetic simulations. [Dissertation, Technische Universität Wien]. reposiTUm. https://doi.org/10.34726/hss.2014.21425 (2014).
4. Klein, D. R. *et al.* Probing magnetism in 2D van der Waals crystalline insulators via electron tunneling. *Science* **360**, 1218–1222 (2018).
5. Nagaosa, N. & Tokura, Y. Topological properties and dynamics of magnetic skyrmions. *Nat. Nanotechnol.* **8**, 899–911 (2013).
6. Braun, H. B. Topological effects in nanomagnetism: from superparamagnetism to chiral quantum solitons. *Adv. Phys.* **61**, 1–116 (2012).
